# Identification of candidate genes and pathways in retinopathy of prematurity by whole exome sequencing of preterm infants enriched in phenotypic extremes

**DOI:** 10.1038/s41598-021-83552-y

**Published:** 2021-03-02

**Authors:** Sang Jin Kim, Kemal Sonmez, Ryan Swan, J. Peter Campbell, Susan Ostmo, R. V. Paul Chan, Aaron Nagiel, Kimberly A. Drenser, Audina M. Berrocal, Jason D. Horowitz, Xiaohui Li, Yii-Der Ida Chen, Kent D. Taylor, Charles Simmons, Jerome I. Rotter, Michael F. Chiang, Imaging and Informatics in Retinopathy of Prematurity (i-ROP) Research Consortium, Michael F. Chiang, Susan Ostmo, Sang Jin Kim, Kemal Sonmez, J. Peter Campbell, R. V. Paul Chan, Karyn Jonas, Jason Horowitz, Osode Coki, Cheryl-Ann Eccles, Leora Sarna, Anton Orlin, Audina Berrocal, Catherin Negron, Kimberly Denser, Kristi Cumming, Tammy Osentoski, Tammy Check, Mary Zajechowski, Thomas Lee, Evan Kruger, Kathryn McGovern, Charles Simmons, Raghu Murthy, Sharon Galvis, Jerome Rotter, Ida Chen, Xiaohui Li, Kent Taylor, Kaye Roll, Jayashree Kalpathy-Cramer, Deniz Erdogmus, Stratis Ioannidis, Maria Ana Martinez-Castellanos, Samantha Salinas-Longoria, Rafael Romero, Andrea Arriola, Francisco Olguin-Manriquez, Miroslava Meraz-Gutierrez, Carlos M. Dulanto-Reinoso, Cristina Montero-Mendoza

**Affiliations:** 1 Department of Ophthalmology, Casey Eye Institute, Oregon Health and Science University, 3375 SW Terwilliger Boulevard, Portland, OR 97239 USA; 2 Department of Ophthalmology, Samsung Medical Center, Sungkyunkwan University School of Medicine, Seoul, Korea; 3 Knight Cancer Institute, Cancer Early Detection Advanced Research Center, Oregon Health and Science University, Portland, OR USA; 4 Department of Medical Informatics and Clinical Epidemiology, Oregon Health and Science University, Portland, OR USA; 5 Department of Ophthalmology and Visual Sciences, Illinois Eye and Ear Infirmary and Center for Global Health, College of Medicine, University of Illinois At Chicago, Chicago, IL USA; 6 The Vision Center, Department of Surgery, Children’s Hospital Los Angeles, Los Angeles, CA USA; 7 Department of Ophthalmology, Keck School of Medicine, Roski Eye Institute, University of Southern California, Los Angeles, CA USA; 8 Associated Retinal Consultants, Royal Oak, MI USA; 9 Department of Ophthalmology, Bascom Palmer Eye Institute, Miller School of Medicine, University of Miami, Miami, FL USA; 10 Columbia University College of Physicians and Surgeons, New York, NY USA; 11 Institute for Translational Genomics and Population Sciences and Department of Pediatrics, The Lunquist Institute At Harbor-UCLA Medical Center, 1124 W Carson Street, Torrance, CA 90502 USA; 12 Department of Pediatrics, Cedars-Sinai Medical Center, Los Angeles, CA USA; 13 Weill Cornell Medical College, New York, NY USA; 14 William Beaumont Hospital, Royal Oak, MI USA; 15 Massachusetts General Hospital, Boston, MA USA; 16 Northeastern University, Boston, MA USA; 17 Asociacion Para Evitar La Ceguera en Mexico (APEC), Mexico City, Mexico

**Keywords:** Retinopathy of prematurity, Medical genomics

## Abstract

Retinopathy of prematurity (ROP) is a vasoproliferative retinal disease affecting premature infants. In addition to prematurity itself and oxygen treatment, genetic factors have been suggested to predispose to ROP. We aimed to identify potentially pathogenic genes and biological pathways associated with ROP by analyzing variants from whole exome sequencing (WES) data of premature infants. As part of a multicenter ROP cohort study, 100 non-Hispanic Caucasian preterm infants enriched in phenotypic extremes were subjected to WES. Gene-based testing was done on coding nonsynonymous variants. Genes showing enrichment of qualifying variants in severe ROP compared to mild or no ROP from gene-based tests with adjustment for gestational age and birth weight were selected for gene set enrichment analysis (GSEA). Mean BW of included infants with pre-plus, type-1 or type 2 ROP including aggressive posterior ROP (n = 58) and mild or no ROP (n = 42) were 744 g and 995 g, respectively. No single genes reached genome-wide significance that could account for a severe phenotype. GSEA identified two significantly associated pathways (smooth endoplasmic reticulum and vitamin C metabolism) after correction for multiple tests. WES of premature infants revealed potential pathways that may be important in the pathogenesis of ROP and in further genetic studies.

## Introduction

Retinopathy of prematurity (ROP) is a retinal vascular disease affecting prematurely born infants. Although there have been many advances in neonatal care and management for ROP, ROP remains a leading cause of childhood blindness throughout the world^1,2^. The most significant risk factors for ROP include low birth weight (BW), early gestational age (GA) and oxygen treatment^3^. However, some high-risk infants with low BW and early GA do not develop ROP, whereas some low-risk infants develop severe ROP including aggressive posterior ROP (AP-ROP)^4–7^. In these infants at phenotypic extremes, a study from our group demonstrated that known clinical risk factors were not significantly associated with development of ROP, suggesting presence of other risk factors for ROP^8^.

The i-ROP consortium includes collaborators from 14 academic institutions throughout the world with the goal of developing better methods for diagnosing, understanding and treating ROP through computer-based image analysis, genetic analysis and biomedical informatics analysis. Over the past 10 years, over 1700 subjects have participated in the research where demographic data, eye exam data, images taken at the eye exams, as well as other systemic health data have been collected and stored in a large data repository. The 100 non-Hispanic Caucasian preterm infants in this study were selected from the i-ROP consortium samples with the goal of building a sample set that is enriched in phenotypic extremes.

Genetic factors have been suggested to predispose to ROP^3,9^. Racial differences in incidence and severity of ROP^3,10,11^, high concordance rate among monozygotic twins^12,13^, and strain differences in animal models of ROP^14–16^ suggest possible roles of genetic factors in ROP. However, the field of ROP genetics is still in its infancy. A number of studies investigated the frequency of specific genetic variants in premature infants with or without ROP^9^. However, few of them showed strong association or were replicated in other populations, although some studies reported promising gene variants^17,18^. Moreover, most studies examined only a few variants from a small number of candidate genes, which were mostly related to retinal angiogenesis^9^. Studies using updated technologies such as genome-wide association studies (GWAS) or next-generation sequencing (NGS) based approaches have not been reported in ROP.

Whole exome sequencing (WES) has revealed novel pathogenic genes or variants especially in Mendelian disorders (e.g. retinitis pigmentosa) and also in multifactorial diseases (e.g. glaucoma^19–22^, age-related macular degeneration^23–25^) in ophthalmology. Also, combining WES and pathway analysis has enabled researchers to find novel biological pathways or polygenic burdens associated with disease in small to moderate-scale sequencing studies^19,26–28^. This study aimed to identify potentially pathogenic genes and biological pathways associated with ROP by analyzing whole exome sequencing data from 100 preterm infants enriched in phenotypic extremes, by analyzing the variants both by rare variant methods and common variant methods.

## Results

### Characteristics of subjects

**Figure 1:**
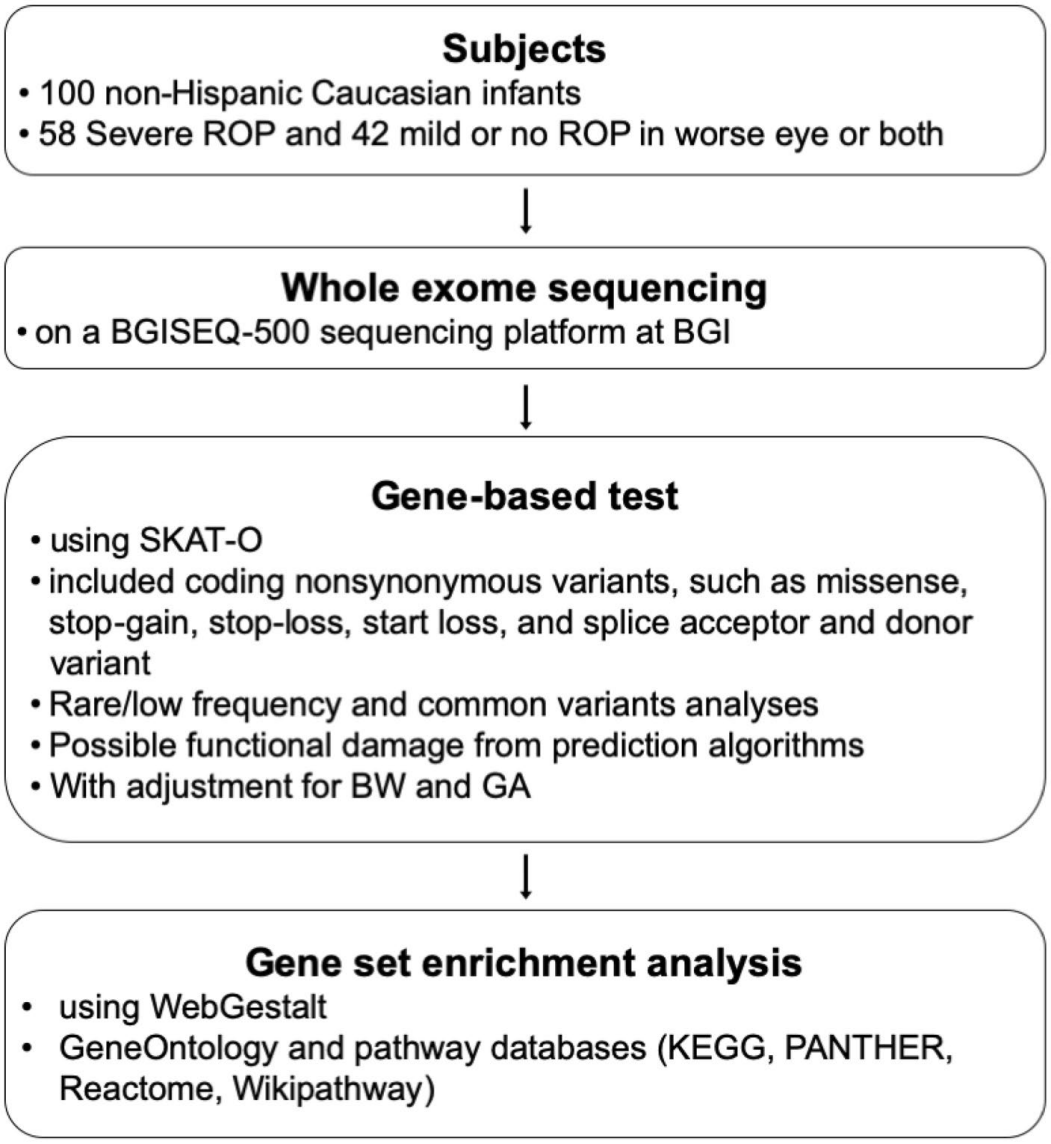
The overall scheme of this study. *ROP* retinopathy of prematurity, *SKAT-O* sequence kernel association optimal unified test, *BW* birth weight, *GA* gestational age, *KEGG* Kyoto Encyclopedia of Genes and Genomes, *PANTHER* Protein ANalysis THrough Evolutionary Relationships.

The overall scheme of this study is shown in Fig. 1. Fifty-eight premature infants with severe ROP (49 type 1 and 9 type 2 or pre-plus) and 42 with mild or no ROP (5 mild and 37 no ROP) in the worse eye or both eyes were included. Characteristics of 100 subjects including demographics, characteristics of ROP and associated morbidities of prematurity are summarized in Table 1. In the severe ROP group, 12 patients with bilateral AP-ROP were included.

**Table 1:** Characteristics of subjects: demographics, retinopathy of prematurity characteristics, and associated morbidities of prematurity.

Parameters	Patient group	
	No or mild ROP	Severe ROP
No. of infants	42	58
Birth weight (g), mean ± SD	995 ± 247	744 ± 256
Gestational age (weeks), mean ± SD	27.6 ± 2.0	25.2 ± 2.0
Gender, male (%)	16 (38)	31 (53)
**Race/ethnicity (%)**		
Non-Hispanic White	100	100
**Lowest zone, no. of eyes (%)**		
Never Developed	72 (86)	0 (0)
Zone I	0 (0)	30 (26)
Zone II or III	12 (14)	86 (74)
**Highest stage of ROP, no. of eyes (%)**		
Stage 0	72 (86)	0 (0)
Stage 1	2 (2)	0 (0)
Stage 2	10 (12)	24 (21)
Stage 3	0 (0)	92 (79)
**Worst plus disease category, no. of eyes (%)**		
Plus	0 (0)	57 (49)
No plus		
Pre-plus	0 (0)	35 (30)
Normal	84 (100)	24 (21)
AP-ROP, No. of eyes (%)	0 (0)	24 (21)
**Associated morbidities of prematurity and management**		
BPD (chronic lung disease) (%)	11 (26)	40 (69)
IVH (%)		
None	36 (85)	36 (62)
Grade I–II	3 (7)	14 (24)
Grade III–IV	3 (7)	8 (14)
Sepsis, overall (%)	5 (12)	29 (50)
Bacterial (%)	3 (7)	26 (45)
Fungal (%)	0 (0)	2 (3)
Unknown type (%)	2 (5)	1 (2)
NEC, surgical (%)	1 (2)	10 (17)

^+^Two sample test of proportion.

*Student two-sample T-test.

^Fisher exact table test.

*ROP* retinopathy of prematurity, *BPD* bronchopulmonary dysplasia, *IVH* intraventricular hemorrhage, *NEC* necrotizing enterocolitis.

### Sequencing results

The mean percentage of mapping on genome was 99.8% and mean sequencing depth on target 149.8 ± 36.8 (Supplemental Table S1). A minimum 1- and tenfold coverage per base was achieved on average for 95.9% and 92.0% of the target region, respectively (Supplemental Table S1). The number of each type of variant is summarized in Supplemental Table S2. Mean number of missense variants per patient was 9283 ± 271.

### Gene-based analysis

Gene-based analysis using SKAT-O, which combines the effects of all rare variants across a gene, with adjustment for BW and GA revealed no genes that reached genome-wide significance (*P* < 2.5 × 10^–6^). From the rare/low-frequency variants analysis, the most strongly associated genes by SKAT-O included *RNF225, PMS1, PRMT3, SPANXD, ANKRD30A* and *THSD4* (Table 2). Among the 263 candidate genes, *CD36, NOX4, NTRK2, IGFBP7*, and *NTF4* were the most strongly associated with ROP (Supplemental Table S3). From the common variants analysis, which analyzes each variant separately, the most strongly associated genes by SKAT-O included *ZNF341, ALG10B, OR4D6, KCNE1,* and *FAM198A* (Table 3).

**Table 2:** Results of rare/low-frequency variants analysis: the 20 most strongly associated genes in 100 non-Hispanic Caucasian preterm infants by SKAT-O.

Rank	Official symbol	Gene name	Entrez gene ID	*P* value	Number of included variants	Number of variant alleles	
						No or mild ROP	Severe ROP
1	*RNF225*	Ring finger protein 225	646882	2.39E−04	1	10	2
2	*PMS1*	PMS1 homolog 1, mismatch repair system component	5378	2.51E−04	5	7	0
3	*PRMT3*	Protein arginine methyltransferase 3	10196	2.61E−04	4	2	2
4	*SPANXD*	SPANX family member D	64648	3.29E−04	2	34	24
5	*ANKRD30A*	Ankyrin repeat domain 30A	91074	3.30E−04	4	16	20
6	*THSD4*	Thrombospondin type 1 domain containing 4	79875	5.04E−04	2	4	0
7	*ATP13A4*	ATPase 13A4	84239	6.10E−04	6	10	0
8	*FGD6*	FYVE, RhoGEF and PH domain containing 6	55785	6.20E−04	4	4	0
9	*DNALI1*	Dynein axonemal light intermediate chain 1	7802	8.26E−04	3	0	3
10	*PIGQ*	Phosphatidylinositol glycan anchor biosynthesis class Q	9091	8.28E−04	3	0	3
11	*OPA1*	OPA1, mitochondrial dynamin like GTPase	4976	8.57E−04	1	5	0
12	*MYO3B*	Myosin IIIB	140469	9.61E−04	4	0	4
13	*ATAD3B*	ATPase family, AAA domain containing 3B	83858	9.72E−04	8	11	3
14	*PCSK6*	Proprotein convertase subtilisin/kexin type 6	5046	1.02E−03	7	11	1
15	*CHML*	CHM like, Rab escort protein 2	1122	1.11E−03	1	0	5
16	*TRIM36*	Tripartite motif containing 36	55521	1.12E−03	3	0	3
17	*PPP4R1*	Protein phosphatase 4 regulatory subunit 1	9989	1.17E−03	4	0	5
18	*TBC1D32*	TBC1 domain family member 32	221322	1.44E−03	5	6	6
19	*CATSPER1*	Cation channel sperm associated 1	117144	1.45E−03	5	1	4
20	*RHBDF2*	Rhomboid 5 homolog 2	79651	1.45E−03	3	3	2

**Table 3:** Results of common variants analysis: the 20 most strongly associated genes in 100 non-Hispanic Caucasian preterm infants by SKAT-O.

Rank	Official Symbol	Gene name	Entrez gene ID	*P* value	Number of included variants	Number of variant alleles	
						No or mild ROP	Severe ROP
1	*ZNF341*	Zinc finger protein 341	84905	8.28E−05	6	29	43
2	*ALG10B*	ALG10B, alpha-1,2-glucosyltransferase	144245	2.82E−04	4	47	63
3	*OR4D6*	Olfactory receptor family 4 subfamily D member 6	219983	3.93E−04	5	77	90
4	*KCNE1*	Potassium voltage-gated channel subfamily E regulatory subunit 1	3753	4.14E−04	1	16	47
5	*FAM198A*	Family with sequence similarity 198 member A	729085	4.97E−04	7	145	216
6	*PI16*	Peptidase inhibitor 16	221476	5.23E−04	2	50	56
7	*CSTF2T*	Cleavage stimulation factor subunit 2 tau variant	23283	5.89E−04	3	11	32
8	*SPANXD*	SPANX family member D	64648	7.71E−04	2	38	34
9	*RNASE11*	Ribonuclease A family member 11	122651	7.88E−04	3	27	70
10	*COG7*	Component of oligomeric golgi complex 7	91949	7.99E−04	3	0	15
11	*NEDD9*	Neural precursor cell expressed, developmentally down-regulated 9	4739	8.37E−04	5	89	146
12	*OBSCN*	Obscurin, cytoskeletal calmodulin and titin-interacting RhoGEF	84033	8.59E−04	80	495	634
13	*PKP3*	Plakophilin 3	11187	9.70E−04	11	177	223
14	*MTPN*	Myotrophin	136319	1.06E−03	1	2	12
15	*TLDC1*	TBC/LysM-associated domain containing 1	57707	1.09E − 03	8	87	118
16	*TNK2*	Tyrosine kinase non receptor 2	10188	1.13E−03	13	88	85
17	*CAPN2*	Calpain 2	824	1.25E−03	5	31	49
18	*FAT1*	FAT atypical cadherin 1	2195	1.29E−03	44	256	305
19	*KEL*	Kell blood group, metallo-endopeptidase	3792	1.33E−03	6	12	4
20	*ZUFSP*	Zinc finger with UFM1 specific peptidase domain	221302	1.48E−03	4	23	42

In order to check if there is confounding between genes that are contributing to prematurity and genes that are contributing to ROP specifically, we compared the twenty genes with the largest associations from the rare variant and common variant analysis sets with the largest gene list for prematurity to date^29^ and found no intersection.

### Gene set enrichment analysis (GSEA)

GSEA identified one pathway (GO:0005790, smooth endoplasmic reticulum) from the rare/low-frequency variants analysis that was significantly enriched after correction for multiple tests (Table 4). GSEA also identified 25 pathways from databases including GO, KEGG, PANTHER, Reactome, and Wikipathways with empirical *P* values of less than 0.05, including the beta2 adrenergic receptor signaling pathway, dopamine receptor mediated signaling pathway, T cell activation, nicotinate/nicotinamide metabolism, and platelet-derived growth factor (PDGF) signaling pathway (Table 4). From the common variants analysis, GSEA identified one significantly enriched pathway (Reactome R-HSA-196836, Vitamin C metabolism) (Table 5). Other pathways with empirical *P* values of less than 0.05 are listed in Table 5.

**Table 4:** Results of rare/low-frequency variants analysis: a list of the most significantly enriched pathways (*P* value < 0.05) from gene set enrichment analysis using WebGestalt.

Accession	Pathway name	*P* value	Adjusted *P* value*
**Gene ontology biological process**			
GO:1903828	Negative regulation of cellular protein localization	4.40E−02	6.42E−01
GO:0002263	Cell activation involved in immune response	3.90E−02	6.43E−01
GO:0044706	Multi-multicellular organism process	2.60E−02	6.50E−01
**Gene ontology cellular localization**			
GO:0005790	Smooth endoplasmic reticulum	5.02E−03	**4.70E**−**02**
GO:0042579	Microbody	8.00E−03	4.46E−01
GO:0044815	DNA packaging complex	4.60E−02	4.70E−01
GO:0005793	Endoplasmic reticulum-Golgi intermediate compartment	8.00E−03	4.80E−01
GO:0031225	Anchored component of membrane	2.80E−02	4.94E−01
GO:0031201	SNARE complex	2.90E−02	5.10E−01
GO:0048770	Pigment granule	1.50E−02	5.75E−01
GO:0044306	Neuron projection terminus	4.20E−02	5.88E−01
**Gene ontology molecular function**			
GO:0000149	SNARE binding	2.00E−03	6.77E−01
GO:0016776	Phosphotransferase activity, phosphate group as acceptor	1.61E−02	7.26E−01
GO:0001085	RNA polymerase II transcription factor binding	1.00E−02	7.69E−01
**KEGG**			
hsa00760	Nicotinate and nicotinamide metabolism: Homo sapiens	1.01E−02	3.73E−01
**PANTHER**			
P04378	Beta2 adrenergic receptor signaling pathway	4.24E−02	2.69E−01
P05912	Dopamine receptor mediated signaling pathway	1.11E−02	3.00E−01
P00053	T cell activation	1.70E−02	3.48E−01
P00047	PDGF signaling pathway	2.80E−02	5.16E−01
**Reactome**			
R-HSA-2151201	Transcriptional activation of mitochondrial biogenesis	3.01E−03	4.67E−01
R-HSA-179419	APC:Cdc20 mediated degradation of cell cycle proteins prior to satisfation of the cell cycle checkpoint	2.11E−02	5.70E−01
R-HSA-453276	Regulation of mitotic cell cycle	1.40E−02	5.74E−01
R-HSA-2262752	Cellular responses to stress	2.50E−02	5.76E−01
**Wikipathways**			
WP481	Insulin signaling	1.90E−02	7.74E−01
WP455	GPCRs, Class A Rhodopsin-like	8.00E−03	7.81E−01

*SNARE* soluble N-ethylmaleimide-sensitive factor attachment protein receptor, *PDGF* platelet-derived growth factor, *APC/C* anaphase-promoting complex, *GPCR* G-protein-coupled receptor.

*By Benjamini–Hochberg procedure.

Numbers in bold indicate the smallest adjusted *p*-values, i.e. most significant pathway enrichments among all rows in the table.

**Table 5:** Results of common variants analysis: a list of the most significantly enriched pathways (*P* value < 0.05) from gene set enrichment analysis using WebGestalt.

Accession	Pathway Name	*P* value	Adjusted *P* value*
**Gene ontology biological process**			
GO:0034367	Macromolecular complex remodeling	8.12E−03	4.61E−01
GO:0048857	Neural nucleus development	3.01E−03	6.05E−01
GO:0060191	Regulation of lipase activity	3.00E−03	6.53E−01
GO:0035902	Response to immobilization stress	6.15E−03	6.79E−01
GO:0002507	Tolerance induction	2.97E−02	7.11E−01
GO:0090077	Foam cell differentiation	1.00E−02	8.03E−01
**Gene ontology cellular localization**			
GO:0032994	Protein–lipid complex	9.02E−03	4.21E−01
GO:0060076	Excitatory synapse	2.53E−02	4.80E−01
GO:0098636	Protein complex involved in cell adhesion	2.80E−02	5.24E−01
GO:1990391	DNA repair complex	2.71E−02	5.61E−01
GO:0042383	Sarcolemma	3.00E−03	5.97E−01
GO:0045178	Basal part of cell	3.10E−02	6.35E−01
**Gene ontology molecular function**			
GO:0005487	Nucleocytoplasmic transporter activity	1.21E−02	7.29E−01
**KEGG**			
hsa04924	Renin secretion: Homo sapiens (human)	2.00E−03	3.87E−01
hsa04930	Type II diabetes mellitus: Homo sapiens (human)	7.00E−03	4.13E−01
hsa03430	Mismatch repair: Homo sapiens (human)	1.22E−02	4.92E−01
**PANTHER**			
P04378	Beta2 adrenergic receptor signaling pathway	4.24E−02	2.69E−01
**Reactome**			
R-HSA-196836	Vitamin C (ascorbate) metabolism	1.15E−02	**4.71E**−**02**
R-HSA-5693616	Presynaptic phase of homologous DNA pairing and strand exchange	0.00E+00	4.77E−01
R-HSA-170670	Adenylate cyclase inhibitory pathway	3.12E−03	4.83E−01
R-HSA-71288	Creatine metabolism	3.35E−03	5.12E−01
R-HSA-997269	Inhibition of adenylate cyclase pathway	4.11E−03	5.19E−01
R-HSA-3656253	Defective EXT1 causes exostoses 1, TRPS2 and CHDS	0.00E+00	5.35E−01
R-HSA-448706	Interleukin-1 processing	1.84E−02	5.41E−01
R-HSA-3656237	Defective EXT2 causes exostoses 2	2.06E−03	5.55E−01
R-HSA-8868766	rRNA processing in the mitochondrion	2.25E−03	5.84E−01
**Wikipathways**			
WP3407	FTO Obesity Variant Mechanism	1.68E−02	8.61E−02
WP3601	Composition of Lipid Particles	3.39E−03	3.44E−01
WP3943	Robo4 and VEGF Signaling Pathways Crosstalk	3.40E−02	3.65E−01
WP1584	Type II diabetes mellitus	7.19E−03	4.84E−01
WP3657	Hematopoietic Stem Cell Gene Regulation by GABP alpha/beta Complex	1.12E−02	5.56E−01
WP3634	Insulin signaling in human adipocytes (normal condition)	3.45E−02	5.93E−01
WP3635	Insulin signaling in human adipocytes (diabetic condition)	2.21E−02	6.29E−01
WP2011	SREBF and miR33 in cholesterol and lipid homeostasis	1.35E−02	6.50E−01
WP3301	MFAP5-mediated ovarian cancer cell motility and invasiveness	3.30E−02	6.62E−01
WP3844	PI3K-AKT-mTOR signaling pathway and therapeutic opportunities	2.51E−02	6.62E−01
WP727	Monoamine Transport	4.32E−02	6.66E−01
WP430	Statin Pathway	2.01E−03	6.66E−01

*EXT1* Exostosin 1, *TRPS2* Trichorhinophalangeal syndrome type 2, *CHDS* chondrosarcoma, *EXT2* Exostosin 2, *Robo4* Roundabout homolog 4, *VEGF* vascular endothelial growth factor, *SREBF* Sterol regulatory element-binding transcription factor, *MFAP5* Microfibril Associated Protein 5, *PI3K* Phosphoinositide 3-kinase, *AKT* Protein kinase B, *mTOR* mammalian target of rapamycin.

*By Benjamini–Hochberg procedure.

Numbers in bold indicate the smallest adjusted *p*-values, i.e. most significant pathway enrichments among all rows in the table.

## Discussion

This study aimed to identify potential genes and pathways associated with ROP by analyzing rare and low-frequency variants from whole exome sequencing data of 100 preterm infants enriched in phenotypic extremes. The key findings from this study are as follows: (1) gene-based analysis with adjustment for BW and GA revealed no genes that reached genome-wide significance; and (2) GSEA identified 2 significantly enriched pathways (smooth endoplasmic reticulum and vitamin C metabolism) that may be important in the pathogenesis of ROP.

Gene-based analysis, which aggregates all the rare variants in each gene, using SKAT-O with adjustment for BW and GA identified many genes with low *P* values, although no genes reached formal genome-wide significance (*P* < 2.5 × 10^–6^). The top most strongly associated genes included several genes of potential interest (Table 2 and 3). *THSD4* (*P* = 5.04 × 10^–4^) encodes thrombospondin type-1 domain-containing protein 4, also known as “A disintegrin and metalloproteinase with thrombospondin motifs-like protein 6” (ADAMTSL-6). ADAMTSL-6, expressed in various tissues including retina (*from FANTOM5 data*), is an extracellular matrix protein that promotes assembly of the fibrillin-1 matrix^30^. Fibrillin-1 controls activation of TGF-β^31,32^, which is an angiogenic activator and has been implicated in retinal vascular diseases such as diabetic retinopathy^33,34^. Therefore, *THSD4* is one potential target for studies on ROP pathogenesis.

*OPA1*, the most common gene mutated in dominant optic atrophy, encodes a dynamin-related GTPase that is necessary for mitochondrial inner membrane fusion and maintenance of mitochondrial architecture^35^. Recently, it was suggested that diabetes resulted in reduced opa1 gene expression and mitochondrial dysfunction in an animal model of diabetic retinopathy (Verma A et al. IOVS 2016;57:ARVO E-Abstract 5446). However, the role of *OPA1* in retinal angiogenesis has not been investigated.

Calpain 2, which is encoded by *CAPN2*, has been known to be involved in neurodegeneration. A recent study showed that inhibition of calpain 2 ameliorated retinal ischemic injury, suggesting that calpain-2 inhibitor might prevent ischemia-induced retinal degeneration^36^. Therefore, *CAPN2* is a potential target for the treatment of ROP.

Among the 263 candidate genes, *CD36* showed the lowest *P* value from SKAT-O test (Supplemental Table S3). *CD36* encodes platelet glycoprotein 4, also known as the thrombospondin receptor. Platelet glycoprotein 4 was suggested to be involved in angiogenesis in various ways with or without mediating thrombospondin^37,38^. Also, as a multi-ligand scavenger receptor, it has been implicated in retina homeostasis^38^. Therefore, CD36 may also be a potential target for future ROP studies.

GSEA identified several pathways that may be important in the pathogenesis of ROP. Identified pathways included the β2 adrenergic receptor signaling pathway, dopamine receptor mediated signaling pathway, T cell activation, PDGF signaling pathway, Robo4 and VEGF signaling pathways crosstalk, and vitamin C (ascorbate) metabolism (Table 4 and 5). A series of studies showed that the β2 adrenergic receptors play a pivotal role in the regulation of vascular endothelial growth factor (VEGF) production and retinal neovascularization^39^. Also, the β1/β2 adrenergic receptor antagonist propranolol showed reduction in VEGF expression, retinal neovascularization and vascular leakage in oxygen induced retinopathy^39^. A recent systematic review on clinical trials which investigated the effect of beta-blockers on ROP concluded that low to moderate quality evidence suggests that prophylactic administration of oral beta-blockers might reduce progression towards stage 3 ROP and decrease the need for treatment^40^.

Retinal dopamine, which is synthesized in and released by subtypes of amacrine and interplexiform cells, and its receptor signaling pathway has not been associated with retinal angiogenesis^41^. However, studies suggested that dopamine mediates diverse functions including retina development, visual signaling, and myopic eye growth^41,42^. Thus, further studies to examine the relationships between dopamine receptor mediated signaling and retinal vascular development or ROP is warranted.

A growing body of evidence supports important roles of inflammation in ROP. Recently, a study showed that regulatory T cells are recruited to the retina in oxygen-induced retinopathy and reduce severe microvascular disease^43^. In this manner, the T cell activation pathway might be related to development of ROP. The PDGF signaling pathway was also important for pericyte viability and the subsequent prevention of VEGF/VEGFR-2 overexpression and angiogenesis in oxygen-induced retinopathy^44^. Although PDGF antagonists have been tried for patients with neovascular age-related macular degeneration with unclear benefits (https://clinicaltrials.gov/ct2/show/NCT01944839; Last accessed on 09/13/2019)^45^, the exact role of PDGF in ROP requires further investigation.

One of the identified pathways (GO:0005790, smooth endoplasmic reticulum) was significantly enriched after correction for multiple tests. In general, smooth endoplasmic reticulum is associated with production of carbohydrate and lipids such as steroid hormones, cholesterol and membrane phospholipids. It also plays an important role in protein modification and intracellular protein transport. However, whether biological processes in smooth endoplasmic reticulum have specific roles in ROP is not known.

A few studies investigated the role of vitamin C in ROP or retinal angiogenesis. An in vitro study showed that vitamin C prevented VEGF-induced increases in endothelial permeability^46^, and retinal level of vitamin C was reduced in the rat model of ROP^47^. However, a randomized controlled trial in 2005 which compared high or low dose supplementation of vitamin C on clinical outcome including ROP showed no significant effects on the development of (any stage) ROP^48^. Further studies to examine the relationships between vitamin C metabolism and ROP is warranted.

This study has several limitations. First, the statistical power was not high because of small sample size (58 severe ROP and 42 mild or no ROP). The small sample size may be one of the reasons why no genes reached genome-wide significance in this study. Second, although we tried to include phenotypic extremes to overcome low statistical power due to small sample size, we could not identify enough phenotypically extreme patients from the i-ROP database. In this study, the severe ROP group included 12 patients with AP-ROP and the remaining patients were selected for highest birthweight in severe ROP group and lowest birthweight in no or mild ROP group. Thus, we believe that our subjects are enriched in phenotypic extremes, which may mean possible enrichment of rare pathogenic variants in our subjects. Third, as a result of the first two limitations, identified association with pathways that are plausible biologically need to be replicated in larger studies.

This study is, to the best of our knowledge, the first NGS-based genetic study in ROP. In this study, we analyzed WES data of 100 premature infants from the large-scale multicenter i-ROP consortium. Although no genes reached genome-wide significance, the results revealed genes and pathways that may be important in development or progression of ROP. Novel genes and pathways may be the targets of future genetic studies such as GWAS.

## Methods

This study was approved by the Institutional Review Board at the coordinating center (Oregon Health and Science University) and at each of 8 study centers (Columbia University, University of Illinois at Chicago, William Beaumont Hospital, Children’s Hospital Los Angeles, Cedars-Sinai Medical Center, University of Miami, Weill Cornell Medical Center, and the Genomics Institute at The Lundquist Institute / Harbor-UCLA Medical Center). This study was conducted in accordance with the Declaration of Helsinki^49^. Written informed consent for the study was obtained from parents of all infants enrolled.

### Subjects

The subjects for this study were selected from participants of the *Imaging and Informatics for ROP (i-ROP)* study, a prospective multicenter cohort study which enrolled preterm infants for ROP screening and collected clinical and imaging data (retinal images obtained using a wide-angle fundus camera [RetCam; Natus Medical Incorporated, Pleasanton, CA]) and blood or saliva samples. In this study, the ROP diagnosis of each eye exam was made by combining clinical exam at each study center and image-based diagnoses by 3 trained graders, as previously described^50^. The *i-ROP* study database was reviewed to identify 100 non-Hispanic Caucasian infants enriched in phenotypic extremes (e.g. AP-ROP, non-LBW infants with severe ROP, LBW infants with no ROP) to maximize variant discovery. Among the 373 non-Hispanic Caucasian infants enrolled between July 2011 and October 2016, patients with AP-ROP in at least one eye were selected first. Remaining cases were selected for lowest birthweight in the case of no or mild ROP (defined as ROP less than type 2 ROP), and highest birthweight in severe ROP (defined as pre-plus, type-2 or type 1 ROP), with an enforced ratio of approximately 4:1:1:4 of no, mild, pre-plus/type-2, and type 1 ROP, respectively. In cases where a patient had a twin in the selected set, the most phenotypically extreme infant of the two was selected. Patients without detailed information on demographics, ROP screening, co-morbidities of prematurity, or imaging data were excluded.

### Whole exome sequencing

Genomic DNA was extracted from peripheral blood samples and whole-exome sequencing was performed at Beijing Genomics Institute (BGI; Hong Kong, China). Briefly, genomic DNA was randomly fragmented and enriched for exome sequences using the SureSelect Human All Exon Kit (Agilent Technologies, Santa Clara, CA, USA) and sequencing was performed on a BGISEQ-500 sequencing platform (BGI; Hong Kong, China). Sequencing-derived raw image files were processed by BGISEQ-500 base-calling Software for base-calling and the sequence data of each sample was generated as paired-end reads^51^.

### Bioinformatics analyses

After removing reads containing sequence adaptors and low-quality reads, reads of each sample were aligned to the reference human genome, Genome Reference Consortium Human Build 37 using Burrows-Wheeler Aligner software. Local realignment around insertions and deletions (InDels) and base quality score recalibration were performed using Genome Analysis Toolkit (GATK), with duplicate reads removed by Picard MarkDuplicates. The HaplotypeCaller of GATK was used to call SNPs. After performing hard-filtering for SNPs and Indels as previously described^52^, the SnpEff tool was used to annotate SNPs (reported in dbSNP v.141) and Indels. The variants were annotated with the allele frequency in 1000 Genomes Project (http://www.internationalgenome.org/)53 or ESP6500 database (http://evs.gs.washington.edu/EVS/), and with prediction algorithms including SIFT^54^, PolyPhen2^55^, MutationAssessor^56^, FATHMM^57^, and MutationTaster^58^.

### Selection of candidate genes

Previously reported candidate genes in ROP and additional genes involving pathways in retinal angiogenesis/vasculogenesis, neuronal development, neuroprotection, and retinal inflammation were selected (n = 164; Supplemental Table S4). Also, additional genes (n = 99) that are related to the 164 candidate genes were identified using Cytoscape with GeneMANIA plugin which finds related genes using large-scale functional association data including protein and genetic interactions, pathways, co-expression, co-localization and protein domain similarity (Supplemental Table S5)^59^.

### Gene-based test

Gene and pathway analyses were run on two subsets of data. First, gene-based testing was performed to detect rare and low-frequency variants with relatively large effect. For the test, variants with a MAF of greater than 0.05 in 1000 Genomes Project and ESP6500 were excluded. In gene-based testing, in order to provide sufficient power, all rare variants within the gene are combined for the statistical analysis. Second, for the common variant analysis, variants with a MAF of ≥ 0.05 in 1000 Genomes Project or ESP6500 were selected. In common variant analysis, each variant is analyzed separately. The qualifying variants for gene-based tests included coding nonsynonymous variants, including missense, stop-gain, stop-loss, start loss, and splice acceptor and donor variants that were predicted to be functionally damaging from at least 1 of the 5 prediction algorithms (SIFT, PolyPhen2, MutationAssessor, FATHMM, and MutationTaster). These qualifying variants in individual genes from the two subsets of data (rare / low-frequency variants and common variants) were subject to sequence kernel association optimal unified test (SKAT-O)^60^ using the genipe (GENome-wide Imputation PipelinE) v.1.4.0 module^61^ with adjustment for BW and GA.

### Gene set enrichment analysis

Gene set enrichment analysis (GSEA) was performed using WebGestalt (WEB-based Gene SeT AnaLysis Toolkit, http://www.webgestalt.org) using two sets of genes from the rare/low-frequency and common variants analyses, respectively^62^. For GSEA, we included Gene Ontology (GO, http://geneontology.org)^63^, KEGG (Kyoto Encyclopedia of Genes and Genomes, http://www.kegg.jp/)^64^, PANTHER database (Protein ANalysis THrough Evolutionary Relationships, http://www.pantherdb.org)^65^, Reactome (https://reactome.org)^66^, and WikiPathways (https://wikipathways.org)^67^. The obtained *P* values were adjusted by Benjamini–Hochberg correction (false discovery rate, *P* < 0.05).

### Conference presentation

Portions of this study were presented at the 2018 ARVO annual meeting in Honolulu, HI, on May 1, 2018.

## Supplementary Information

Supplementary Information 1.

Supplementary Information 2.
