# Supplementary material for: Identification of candidate genes and pathways in retinopathy of prematurity by whole exome sequencing of preterm infants enriched in phenotypic extremes
Source: Sci Rep. 2021 Mar 2;11:4966. doi: 10.1038/s41598-021-83552-y (PMC7925531; doi:10.1038/s41598-021-83552-y)
Supplement: Supplementary file 1 — Supplementary Information 1. [file 41598_2021_83552_MOESM1_ESM.docx]

**Identification of candidate genes and pathways in retinopathy of prematurity by whole exome sequencing of preterm infants enriched in phenotypic extremes**

Sang Jin Kim,1,2* Kemal Sonmez,3* Ryan Swan, 4J. Peter Campbell, 1Susan Ostmo, 1R. V. Paul Chan, 5Aaron Nagiel, 6Kimberly A. Drenser, 7Audina M. Berrocal, 8Jason D. Horowitz, 9Xiaohui Li, 10Yii-Der Ida Chen, 10Kent D. Taylor, 10Charles Simmons, 11Jerome I. Rotter, 10†Michael F.

Chiang, 1,4†for the Imaging and Informatics in Retinopathy of Prematurity (i-ROP) Research

Consortium

***Supplemental Table S1. Depth of coverage (n=100 patients)***

|  | Effective sequences on target (Mb) | Capture specificity (%) | Mapping rate on genome (%) | Average sequencing depth on target | Fraction of target covered | | | |
| --- | --- | --- | --- | --- | --- | --- | --- | --- |
|  |  |  |  |  | ≥1x (%) | ≥4x (%) | ≥10x (%) | ≥20x (%) |
| Mean number per patient | 11,172 | 59.4 | 99.8 | 149.8 | 95.9 | 93.4 | 92.0 | 90.8 |
| Standard deviation | 2,745 | 3.1 | 0.5 | 36.8 | 6.8 | 10.9 | 12.5 | 13.1 |
| Median | 11,365 | 58.4 | 99.9 | 152.4 | 99.9 | 99.7 | 99.3 | 98.3 |

***Supplemental Table S2. Types of coding SNPs (n=100 patients)***

|  | synonymous | missense | stop gain | stop loss | start loss | splicing |
| --- | --- | --- | --- | --- | --- | --- |
| Mean number per patient | 10,114 | 9,283 | 67 | 31 | 17 | 60 |
| Standard deviation | 329 | 271 | 6 | 2 | 3 | 5 |
| Median | 10,057 | 9,232 | 66 | 31 | 17 | 61 |

***Supplemental Table S3.* The 20 most strongly associated genes (P<0.05) by SKAT-O among the 263 candidate genes**

Align rows horizontally to improve ease of reading

| **rank** | **Official Symbol** | **Gene name** | **Entrez gene ID** | **P value** | **Number of included variants** | **Number of variant alleles** | |
| --- | --- | --- | --- | --- | --- | --- | --- |
|  |  |  |  |  |  | **No or mild ROP** | **Severe ROP** |
| 1 | *CD36* | CD36 molecule | 948 | 1.76E-03 | 4 | 3 | 1 |
| 2 | *NOX4* | NADPH oxidase 4 | 50507 | 6.56E-03 | 3 | 3 | 1 |
| 3 | *NTRK2* | neurotrophic receptor tyrosine kinase 2 | 4915 | 6.83E-03 | 3 | 0 | 3 |
| 4 | *IGFBP7* | insulin like growth factor binding protein 7 | 3490 | 1.11E-02 | 1 | 0 | 1 |
| 5 | *NTF4* | neurotrophin 4 | 4909 | 1.12E-02 | 1 | 0 | 1 |
| 6 | *GPR116* | adhesion G protein-coupled receptor F5 | 221395 | 1.88E-02 | 1 | 4 | 2 |
| 7 | *ANXA2* | annexin A2 | 302 | 2.07E-02 | 1 | 1 | 0 |
| 8 | *IL6ST* | interleukin 6 signal transducer | 3572 | 2.41E-02 | 1 | 0 | 1 |
| 9 | *PDGFA* | platelet derived growth factor subunit A | 5154 | 2.41E-02 | 1 | 0 | 1 |
| 10 | *WNT16* | Wnt family member 16 | 51384 | 2.65E-02 | 1 | 1 | 0 |
| 11 | *WNT10B* | Wnt family member 10B | 7480 | 2.84E-02 | 2 | 0 | 4 |
| 12 | *NCAM1* | neural cell adhesion molecule 1 | 4684 | 2.85E-02 | 6 | 7 | 10 |
| 13 | *TF* | transferrin | 7018 | 3.05E-02 | 4 | 2 | 11 |
| 14 | *FZD4* | frizzled class receptor 4 | 8322 | 3.71E-02 | 4 | 1 | 5 |
| 15 | *CD40* | CD40 molecule | 958 | 3.93E-02 | 1 | 0 | 1 |
| 16 | *EGFR* | epidermal growth factor receptor | 1956 | 3.93E-02 | 1 | 0 | 1 |
| 17 | *GFRA3* | GDNF family receptor alpha 3 | 2676 | 3.93E-02 | 1 | 0 | 1 |
| 18 | *SEMA3E* | semaphorin 3E | 9723 | 3.93E-02 | 1 | 0 | 1 |
| 19 | *WNT10A* | Wnt family member 10A | 80326 | 4.19E-02 | 6 | 4 | 7 |
| 20 | *FLT4* | fms related tyrosine kinase 4 | 2324 | 4.64E-02 | 5 | 10 | 14 |
